# Prevalence, Causes and Social Factors of Visual Impairment among Chinese Adults: Based on a National Survey

**DOI:** 10.3390/ijerph14091034

**Published:** 2017-09-08

**Authors:** Chao Guo, Zhenjie Wang, Ping He, Gong Chen, Xiaoying Zheng

**Affiliations:** 1 Institute of Population Research, Peking University, Beijing 100871, China; chaoguo@pku.edu.cn (C.G.); zhenjie.wang@pku.edu.cn (Z.W.); pkuheping@pku.edu.cn (P.H.); chengong@pku.edu.cn (G.C.); 2 APEC Health Science Academy, Peking University, Beijing 100871, China

**Keywords:** visual impairment, blindness, low vision, China, prevalence, cause, social factors

## Abstract

Visual impairment has become a global challenge, especially for developing countries. This study aims to estimate the prevalence, causes and social factors of visual impairment among Chinese adults. Data were from a nationally representative population-based cross-sectional study. The study population were 1,909,199 non-institutionalized adults aged 18 years and older in mainland China. In the survey, low vision and blindness were checked by ophthalmologists according to the WHO best-corrected visual acuity (BCVA) criteria. Population weighted numbers and prevalence of low vision and blindness with 95% confidence intervals (CIs) were estimated where appropriate. Multivariable logistic regression analysis was used to identify the social factors of visual impairment. The weighted prevalence of visual impairment was 17.17 (95% CI, 16.84–17.50) per 1000 Chinese adults aged 18 years and older. Cataract (57.35%), disorders of choroid and retina (9.80%), and disorders of cornea (6.49%) contributed more than 70 percent to the visual impairment in Chinese adults. Older age groups, young or middle-aged male adults, female elders, illiterate, rural dwellers, non-eastern residents, singles, unemployment, and from family with lower income were associated with visual impairment. More efforts are warranted to enhance treatment and rehabilitation among people with eye disorders to prevent visual impairment.

## 1. Introduction

Visual impairment has become a global challenge, especially for developing countries [1]. In 2010, the World Health Organization estimated that there were 285 million people living with visual impairment worldwide, and 90% lived in low-and middle-income countries [1,2]. Visual impairment is also one of the strongest risk factors for functional status decline in community-living people [3]. People with visual impairments are at high risk of disabilities due to their difficulties in physical activities and social participation [4,5].

A number of previous studies have reported the prevalence and indicated that cataract, glaucoma, age-related macular degeneration (AMD), and corneal opacities, etc. were the main causes of visual impairment in the West and Asian countries [6,7,8,9,10,11]. Visual impairment is also influenced by socioeconomic factors including age, gender, education status, residence area, and so on [9,10,11,12,13,14,15]. As the most populous country, China has an estimated number of 84.6 million persons living with various impairments or disabilities [16]. There were some observational studies on visual impairment conducted in China [17,18,19]. For example, Li et al. reported that the prevalence of visual impairment in Henan province of China was 1.51%, and the prevalence in females and rural residents were significantly higher [20]. Zhao et al. also identified that visual impairment was associated with older age, female gender, lack of education, and geographic area [17].

However, most of the existing studies were conducted in local regions with limited sample size, which could not provide sufficient information on visual impairment throughout the country. Thus the exploration of the factors of visual impairment was not completely attempted. The present study was designed to estimate the prevalence and identify the social factors of visual impairment among Chinese adults based on a nationally representative data.

## 2. Methods

### 2.1. Samples and Interview Procedures

Data were obtained from the second China National Sample Survey Disability (CNSSD), conducted from 1 April 2006 to 31 May 2006, which is the most recent nationally representative survey on disability in China. The survey was approved by the State Council of China (No. 20051104) and conducted according to legal guidelines governed by the Statistical Law of the People’s Republic of China (1996 Amendment). All respondents provided consent to participate in the survey and to undergo clinical examinations for diagnoses. Experts from the National Bureau of Statistics of China, the China Federation of Disabled Persons, and the Division of Statistics of the United Nations reviewed the survey protocol and questions [16]. Details of the survey protocol and implementation were described elsewhere [21,22,23]. The target population of the survey was the non-institutionalized community population (accounting for 99.8% of the total population). Following standard procedures for complex samples, multistage stratified random cluster sampling with probability proportional to size was used to get the nationally representative samples. The survey sampled a total of 2.6 million samples from 5964 communities/areas, 2980 towns/townships and 734 counties of 31 provinces in mainland China, representing 1.9 per 1000 non-institutionalized inhabitants of China.

The survey involved more than 20,000 interviewers, 50,000 survey assistants, as well as 6000 doctors of various specialties. After the strict training at national and province levels according to the standard made by the expert committee of the survey, interviewers collected pre-survey information about the number of households, population, and suspected people with impairment in the sampling community before 25 March 2006. During the formal survey, all family members of the selected households were interviewed. After the basic information of the households was collected, a screening questionnaire for various physical and mental problems was conducted by trained field interviewers (Table S1). The sensitivity and specificity of the questionnaire are 98.4% and 91.1%, respectively. Those who responded “yes” to any of the disability questions were then further tested sequentially by designated physicians to confirm a final diagnosis, assess the severity of the impairment, and confirm its primary causes [16]. Definitions and classification of disabilities were established by the Expert Committee of CNSSD, and were based on the International Classification of Functioning, Disability, and Health (ICF) [5]. In order to quality for disability identification, 99 communities were random selected from the national samples to double check the missing report of disability [16,24]. The age and sex structures of the participants were both similar to the national population by comparing with the national 1% population survey in 2005 [25].

### 2.2. Measures

A “visual impairment” in the survey refers to poor vision and/or constriction of the visual field in the better-seeing eye from an uncorrectable cause, affecting a person’s daily life and social participation. In our survey, visual impairment consists of two categories: blindness and low vision. Those responded “yes” to the question “Do you or any of your family members have any eyesight problem (cannot see clearly or cannot see at all)?” were further tested sequentially by designated ophthalmologists to confirm the visual disability. Visual impairments were diagnosed according to the WHO best–corrected visual acuity (BCVA) criteria (low vision: 0.05 ≤ BCVA < 0.3; blindness: no light perception ≤ BCVA < 0.05, visual field less than 10 degrees; the better-seeing eye) [25,26]. The visual acuities and visual fields (if necessary) of both eyes were checked. The diagnosis was subject to the eye with the better visual acuity, if the visual acuities of eyes were different [27].

The visual acuity was measured by ophthalmologists using the Standard Logarithmic Visual Acuity Chart (GB 11533-1989) at a distance of 2.5 m. Visual acuity was recorded as the smallest line read with 1 or no error, and tested by counting fingers, hand movements, and light perception (or no light perception for those unable to read the top line at 0.5 m). Each eye was measured separately. Participants regularly using eye glasses for correction wore them during the examinations. Examinations were conducted in a well-lit bright locale, with no direct sunlight or shadows [27].

Visual field was checked for those with “no visual impairment” according to visual acuity, but suspected of having one because of <10° visual field. The visual field was measured each eye separately. A field card was designed for use at a distance of 33 cm from the patients' eyes. Each card consisted of a central fixation point with two circular areas of 5° and 10° in diameter, respectively. While fixing gaze on a point in the center of the card, participants (at least one eye) with <10° visual field were identified as blindness, regardless of the visual acuity [27].

Root causes of visual impairment were diagnosed by ophthalmologists with at least 10 years of clinical experience in province-level hospitals or 5 years of clinical experience in county-level hospitals, trained by strict and unified standard. Flashlight, occluder, stick, pinhole lens, lens-arranging, slit lamp and ophthalmoscope etc. were used to exam the anterior and posterior segments of eye. These causes included, but were not limited to, hereditary (eye disease caused by clear hereditary reasons) and congenital abnormalities (ocular structure and function changes caused by congenital reasons, generally found within one year after birth), injury (eye disease caused by injuries), toxicosis (ocular dysfunction caused by drugs or chemical substances), and acquired eye diseases including cataract, glaucoma, trachoma, disorders of cornea, optic neuropathy, disorders of choroid and retina, ametropia, amblyopia and unknown reasons [27].

Age at the time of the survey was set as continuous and we further categorized it as young adults (18–39 years), middle-aged adults (40–64 years) or elders (65 years and older) [28]. Survey respondents were also categorized by gender (male or female), illiterate (yes or no, according to if the participant was able to read or write at 1500 words not including braille), residence (rural areas or urban areas, according to the division of urban and rural areas that the participants living in the survey time by the government), region (east, central or west, according to the geographic division of the provinces that the participants living in the survey time by the government), marital status (unmarried, married, or divorced or widowed), employment (yes or no, according to if the participant was employed at least 1 h with payment during the week before the survey time), annual family income per capita by residential area (>national average or ≤national average, family income meant the total income of all the family members in a household during 2005 and was categorized based on the national average by residential area). 

### 2.3. Statistical Analysis

Allowing for the complex sampling design, we constructed sample weights using standard weighting procedures calculating the inverse probability of inclusion for an individual survey respondent in the multistage sampling frame [29]. Hierarchy data analysis was performed using SAS version 9.1 (SAS Institute, Inc., Cary, NC, USA) because survey design was multi stage cluster sampling, and unequal weighting were used to calculate population-weighted results for the national population in 2006. Population weighted numbers and prevalence of low vision, blindness and visual impairment, with 95% confidence intervals (CIs), for the overall population and for different population segments were estimated where appropriate. The Chi-square test was used for the difference of low vision, blindness and visual impairment within the demographic and socio-economic variables. The Taylor series linearization method was used to estimate the standard errors of proportions for cross-tabulation tables, allowing for both first-stage cluster and stratum variance and corresponding 95% Confidence Interval (CI) [30]. Multivariable logistic regression analysis was used to calculate the adjusted odds ratios (ORs) and 95% confidence interval (CI) of visual impairment relative to those without visual impairment. Demographic and socio-economic variables (age group, gender, education, residence, region, marital status, employment and annual family income per capita) were included in the model as the independent variables. A two-sided *p*-value < 0.05 was set as statistically significance. 

## 3. Results

### 3.1. Characteristics of Samples

In the survey, 2,526,145 persons in 771,797 households were interviewed. The missing report rate of disability in the participants was 1.12 per thousand persons according to the double-checking after the survey. Due to the objective of our study, only information of respondents aged 18 years and older was analyzed. The study population comprised 1,909,199 non-institutionalized adults (≥18 years of age), equivalent to a weighted total of 984,698,518. Among them 43.84% aged 40–64 years, males accounted for 49.74% of the samples, and 67.57% of the respondents lived in rural areas. The distributions of the education level, region, marital status, employment, and annual family income per capita of study population were shown in Table 1.

### 3.2. Prevalence of Visual Impairment among Chinese Adults

The weighted number of Chinese adults aged 18 years and older with visual impairment was estimated to be 16.9 million, and one third of them were blind. The weighted prevalence of visual impairment was 17.17 (95% CI, 16.84–17.50) per 1000 Chinese adults. The weighted prevalence of low vision and blindness were 11.31 (95% CI, 11.05–11.56) per 1000 persons and 5.87 (95% CI, 5.70–6.03) per 1000 persons, respectively

**Figure 1:**
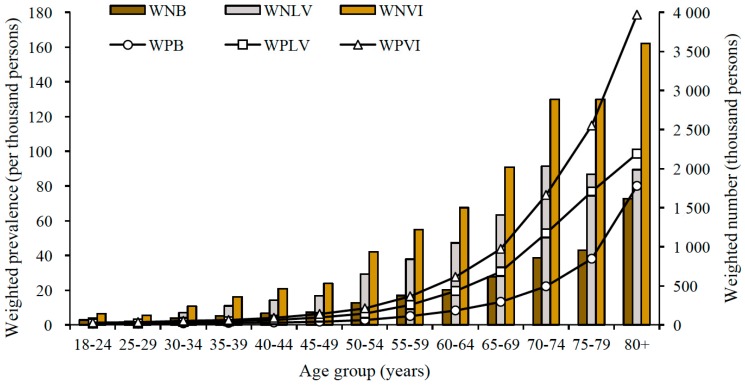
The estimated number and weighted prevalence of low vision, blindness, and visual impairment among Chinese adults, by age group. Note: WNB = weighted number of blindness; WNLV = weighted number of low vision; WNVI = weighted number of visual impairment; WPB = weighted prevalence of blindness; WPLV = weighted prevalence of low vision; WPVI = weighted prevalence of visual impairment.

We calculated the prevalence of low vision, blindness, and visual impairment demographically and socioeconomically (Table 2). The estimated number and weighted prevalence of low vision, blindness, and visual impairment all increased significantly with age group (Figure 1). We also observed the higher weighted prevalence of low vision, blindness, and visual impairment among those who were females, illiterate, rural residents, non-eastern dwellers, divorced or widowed, unemployed, and with lower annual family income per capita (Table 2).

### 3.3. Causes of Visual Impairment among Chinese Adults

The main causes of visual impairment were cataract, disorders of choroid and retina, and disorders of cornea in Chinese adults. For adults aged 18–39 years, the leading cause of visual impairment was hereditary or congenital abnormality, followed by disorders of choroid and retina. The leading causes of visual impairment for adults aged 40–64 years, and 65 years and older, were both cataract (Table 3).

### 3.4. The Social Factors of Visual Impairment among Chinese Adults

The OR of visual impairment increased by 12%, 6% and 9% for each additional year of age among adults aged 18–39 years, 40–64 years, and 65 years and older, respectively. The association between gender and visual impairment differed by age groups. Compared with males, females were at lower visual impairment risk among adults aged 18–64 years, but at higher risk among those aged 65 years and older. Rural residents, non-eastern dwellers, the illiterate, the singles, the unemployed, and those with lower annual family income per capita were at a higher risk of visual impairment than their counterparts among adults in all the three age groups (Table 4).

## 4. Discussion

Data for this study were gleaned from representative samples of the most recent nationwide population–based survey on visual impairment in China. Results demonstrate that a significant number of Chinese adults were living with visual impairment, i.e., 11.1 million low vision and 5.8 million blindness, based on the WHO criteria. Cataract was the leading cause of both low vision and blindness in China.

Previous studies reported that the prevalence of low vision and blindness based on the WHO BCVA criteria in China were 4.9–5.3% and 1.0–1.9% among adults aged 40 years and older [17,18,19], respectively. The prevalence of visual impairment reported in the United States, Qatar and Upper Egypt and were 2.76% [6], 10.22% [11] and 15.7% [15], respectively. These results are all higher than our findings. However, the nationwide prevalence of visual impairment (1.7%) in our finding is almost consistent with the result in Japan (1.3%), an Asian country and has the similar culture and population aging status with China [8]. The lower prevalence in our study may be explained by the differences in survey sites, study populations, and definitions of low vision and blindness. Most previous studies were conducted in older population or a certain city or county in China, the results can only represent the specific population or local situation. Some studies centered on rural residents and the older adults, such as the study in Harbin [18] and Nine-Province Eye Study [17]. In the current study, the prevalence of visual impairment among rural residents and elders was significantly higher than that among urban population and younger adults, so it is reasonable for the higher results in these studies. However, as the most populous country, China contributed more than a quarter of the global visual impairment [31]. The large number of people with visual impairment according to our study indicated a huge national health burden for the government and society.

Consistent with previous studies [12,18,19], cataract was the leading cause of visual impairment in China and its contribution related with age. Other causes of visual impairment found in the current study were also supported by previous studies. For examples, in the Taizhou Eye Study, the second and third leading causes of visual impairment—myopic macular degeneration (MMD) and age-related macular degeneration (AMD)—are included in the second leading cause in our study—disorders of choroid and retina. In addition, the leading cause of visual impairment among young adults aged 18–39 years was founded quite different from that among the older ones, i.e., hereditary and congenital abnormalities accounted for more than a quarter of visual impairment causes. For most of the previous studies focused on adults aged 40 years and older, our findings provided supplemental information for the causes of visual impairment in young adults.

Unsurprisingly, our findings indicated that increased likelihood of visual impairment was commonplace among those who were older, rural dwellers, non-eastern residents, illiterate, singles, unemployed or from low income families, which is consistent with previous studies [13,14,17,20]. According to a previous study, ageing is the most common independent factor of visual impairment [32]. As the age increases, the function of body become poorer, and people can suffer more and more age-related eye diseases, such as cataract, AMD, and diabetic retinopathy, which are also main causes of visual impairment [32]. Other associated factors, people who cannot read or write, living in rural areas or non-eastern regions, singles, unemployment and lower family income, indicated a poorer socioeconomic status (SES), i.e., the lack of concept on health, therapy and rehabilitation, and the barriers and inequality of access to the healthcare services [33]. SES and visual impairment are inter-related. Previous studies show that visual impairment has always been associated with poverty and low socioeconomic status [14,17,33], whereas visual impairment can lead to financial insecurity and social isolation even in affluent countries [34].

Our findings showed that the ORs for lower SES including illiterate, rural residents, divorced or widowed subjects, unemployed, were higher in the youngest group, which indicated a stronger relationship between lower socioeconomic status (SES) and visual impairment in the young adults. This is consistent to previous reports on the narrowing of health differences by SES at older ages [35]. It may be explained by the later-life reductions in impact of exposure to psychosocial risk factors contributing to poor health, the higher impact of biological deterioration in determining health compared with the significance of social factors in old age, as well as the higher sensitivity of the inter-relationship between health (such as wealth and recent family income) and SES for the working-age population [36,37,38]. 

Generally, it was observed in previous studies among older adults (usually 40 years and older) in China and other nations that females were at a higher risk of visual impairment [9,10,11,17], and only the Beijing Eye Study reported that visual impairment was not associated with gender (*p* = 0.76) [39]. Uniquely, in the present study, females were at a lower visual impairment risk than males among adults aged 18–64 years, although the OR of visual impairment was also higher in females than males among those aged 65 years and older. For most of the previous studies focused on older adults, it is a unique finding. According to the statistic of discharged patients of eye diseases in health institutions and hospitals in 2006 by the National Health and Family Planning Commission of China, the proportion of young patients under 45 years of age in males (28.6%) was much higher than that in females (19.7%) [40]. This indicated a larger number of admitted male patients and a higher rate of eye diseases among young males than females, with the hypothesis of the same discharge rate. However, given the lack of more direct information, further studies and evidences on the gender disparity in visual impairment especially among the young and middle-aged adults are needed.

Meanwhile, with the development of society and economy in China, the barriers to access to eye healthcare services in females due to the gender inequality are gradually reducing [41], and the accumulative effect of barriers happened in the past decades may be more visible in the health status of the older females. Additionally, facing the pressure from the stressful work and life in the competitive society in China today, young Chinese males were more likely to neglect their eye problems and tend to less use healthcare service than females, which may result in the higher risk of visual impairment in the young males in China as well [42,43].

The current study has several limitations. Firstly, the social factors identified in this study may not imply causality because of using a cross-sectional survey. Future prospective studies are needed to evaluate how to prevent visual impairment among Chinese adults. Secondly, there may be an underestimate of visual impairment due to the old definition of visual impairment (WHO BCVA) used in the survey and the possible missing in the screening when the problems were not considered serious enough by participants. However, it is difficult to take health examine for more than 2 million participants for a national survey, and the missing report rate of impairment in the participants was low enough (1.12 per thousand persons) according to the double-checking after the survey. In addition, the lack of detailed causes category information of visual impairment makes it difficult to compare between surveys. Regardless of these limitations, based on the fact that the study was based on a large, representative population-based sample covering all provincial areas of China, this study provides a new and broader understanding of low vision and blindness, and pointed the potential social factors of visual impairment at the national level for the first time. Meanwhile, the visual impairment information of young adults that was not much involved in previous studies was provided in the present study.

## 5. Conclusions

In conclusion, our findings indicate that a considerable number of Chinese adults are suffering from visual impairment and a relatively exacerbating situation for those with lower socio-economic status. These results are closer to previous studies from other countries although our nationwide prevalence differ from previous local studies in China. The findings may help increase the awareness of visual impairment by general public and policy makers. Faced with such a stern fact of visual impairment, more efforts are warranted to enhance treatment and rehabilitation among people with eye disorders to prevent visual impairment and improve the lives of people with visual impairment.

## Figures and Tables

**Table 1:** Participants characteristics.

Variables	Sample Number (Persons)	Weighted Number (Million Persons)	Proportion (%)
Total	1,909,199	985	100
Age group (years)			
18–39	822,041	423	42.95
40–64	836,406	432	43.84
65+	250,752	130	13.21
Gender			
Male	949,955	490	49.74
Female	959,244	495	50.26
Illiterate			
Yes	302,635	160	16.25
No	1,606,564	825	83.75
Residence			
Rural	1,229,862	665	67.57
Urban	679,337	319	32.43
Region			
East	769,169	401	40.73
Central	558,169	317	32.2
West	581,861	267	27.07
Marital status			
Unmarried	227,393	115	11.66
Married	1,523,107	788	80.03
Divorced or widowed	158,699	82	8.31
Employment			
Yes	1,367,709	719	72.99
No	541,490	266	27.01
Annual family income per capita by residential area *			
>national average	462,517	233	23.69
≤national average	1,446,682	751	76.31

* The average income was 11,759 RMB for urban residents and 3578 RMB for rural residents.

**Table 2:** The prevalence of low vision, blindness, and visual impairment among Chinese adults.

Variables	Low Vision		Blindness		Visual Impairment	
	WN (Thousand Persons)	WP (per Thousand Persons)	WN (Thousand Persons)	WP (per Thousand Persons)	WN (Thousand Persons)	WP (per Thousand Persons)
Total	11,132	11.31	5777	5.87	16,909,201	17.17
Age group ***						
18–39 years	552	1.31	313	0.74	864,888	2.05
40–64 years	3229	7.48	1418	3.29	4,647,278	10.76
65+ years	7351	56.53	4046	31.12	11,397,035	87.65
Gender ***						
Male	4500	9.19	2,145	4.38	6,644,443	13.57
Female	6633	13.40	3632	7.34	10,264,758	20.74
Illiterate ***						
Yes	6183	38.64	3940	24.63	10,123,566	6.33
No	4949	6.00	1837	2.23	6,785,635	8.23
Residence ***						
Rural	8393	12.61	4733	7.11	13,125,746	19.73
Urban	2739	8.58	1044	3.27	3,783,455	11.85
Region						
East	4050	10.10	1828	4.56	5,878,342	14.66
Central	3549	11.19	1983	6.26	5,532,382	17.45
West	3533	13.25	1965	7.37	5,498,477	20.63
Marital status ***						
Unmarried	354	3.08	389	3.38	742,573	6.46
Married	6541	8.30	27,835	3.53	9,324,121	11.83
Divorced or widowed	4238	51.79	2605	31.83	6,842,507	83.62
Employment ***						
Yes	3081	4.29	782	1.09	3,863,212	5.38
No	8050	30.27	4995	18.78	13,045,990	49.04
Annual family income per capita by residential area ***						
>national average	1470	6.30	643	2.76	2,113,277	9.05
≤national average	9663	12.86	5133	6.83	14,795,924	19.69

Note: WN and WP were short for weighted number and weighted prevalence for the national population in 2006. Wald χ^2^ test was used for the difference of low vision, blindness and visual impairment within the demographic and socio-economic variables; *** *p* < 0.001 for all the results in every column for the highlighted variable.

**Table 3:** Causes of visual impairment among Chinese adults.

Causes	Weighted Number (Thousand Persons) and Proportion (%) of Causes			
	Total Adults	Young Adults	Middle-Aged Adults	Elders
Cataract	9697 (57.35)	61 (7.09)	1401 (30.14)	8235 (72.25)
Disorders of choroid and retina	1657 (9.8)	108 (12.44)	744 (16.01)	805 (7.06)
Disorders of cornea	1097 (6.49)	65 (7.47)	410 (8.82)	623 (5.47)
Glaucoma	817 (4.83)	28 (3.18)	277 (5.95)	513 (4.5)
Hereditary and congenital abnormalities	722 (4.27)	264 (30.54)	340 (7.31)	118 (1.04)
Optic neuropathy	707 (4.18)	81 (9.41)	342 (7.37)	283 (2.48)
Ametropia	656 (3.88)	101 (11.63)	386 (8.31)	169 (1.49)
Injury	333 (1.97)	52 (6.03)	190 (4.09)	90 (0.79)
Trachoma	179 (1.06)	147 (0.02)	52 (1.11)	127 (1.11)
Amblyopia	162 (0.96)	55 (6.36)	85 (1.82)	24 (0.21)
Toxicosis	15 (0.09)	725 (0.08)	11 (0.24)	3 (0.03)
Others	443 (2.62)	22 (2.5)	210 (4.53)	211 (1.85)
Unknown reasons	423 (2.5)	28 (3.26)	200 (4.3)	197 (1.72)
Total	16,909 (100)	865 (100)	4647 (100)	113,975 (100)

Note: The number and proportion were the weighted results for the nation population in 2006.

**Table 4:** The social factors associated with visual impairment among Chinese adults.

Variables	Young Adults		Middle-Aged Adults		Elders	
	Crude OR (95% CI)	Adjusted OR (95% CI)	Crude OR (95% CI)	Adjusted OR (95% CI)	Crude OR (95% CI)	Adjusted OR (95% CI)
**Age**	1.05 (1.04–1.06)	1.12 (1.10–1.13)	1.11 (1.11–1.12)	1.06 (1.06–1.07)	1.11 (1.11–1.12)	1.09 (1.09–1.09)
**Gender (ref = Male)**						
**Female**	0.75 (0.68–0.83)	0.50 (0.44–0.56)	1.18 (1.13–1.23)	0.83 (0.78–0.87)	1.69 (1.64–1.75)	1.15 (1.11–1.20)
**Illiterate (ref = No)**						
**Yes**	11.59 (10.2–13.16)	9.19 (7.87–10.74)	3.14 (2.99–3.3)	1.92 (1.82–2.03)	2.54 (2.44–2.63)	1.47 (1.40–1.54)
**Residence (ref = Urban)**						
**Rural**	1.65 (1.44–1.89)	2.31 (1.95–2.73)	1.53 (1.43–1.63)	2.06 (1.92–2.22)	1.77 (1.67–1.88)	1.71 (1.61–1.81)
**Region (ref = East)**						
**Central**	1.69 (1.46–1.95)	1.48 (1.28–1.72)	1.69 (1.58–1.82)	1.54 (1.43–1.65)	1.06 (1–1.13)	1.02 (0.96–1.08)
**West**	1.97 (1.71–2.26)	1.51 (1.30–1.75)	1.9 (1.77–2.03)	1.59 (1.48–1.70)	1.29 (1.22–1.37)	1.29 (1.21–1.36)
**Marital status (ref = Married)**						
**Unmarried**	1.42 (1.27–1.6)	2.48 (2.07–2.97)	6.17 (5.6–6.79)	4.21 (3.80–4.67)	2.43 (2.14–2.76)	2.43 (2.12–2.79)
**Divorced or widowed**	3.65 (2.84–4.7)	2.46 (1.90–3.18)	2.67 (2.5–2.85)	1.37 (1.28–1.47)	2.31 (2.24–2.39)	1.19 (1.15–1.23)
**Employment (ref = Yes)**						
**No**	4.19 (3.77–4.66)	8.13 (7.01–9.41)	3.64 (3.46–3.83)	3.52 (3.30–3.75)	3.21 (3.02–3.41)	2.19 (2.06–2.33)
**Annual family income per capita by residential area (ref = “>national average”)**						
**≤national average**	2.86 (2.4–3.41)	2.17 (1.82–2.6)	2.58 (2.41–2.77)	2.10 (1.95–2.26)	1.53 (1.45–1.62)	1.44 (1.37–1.52)

## Supplementary Materials

The following are available online at www.mdpi.com/1660-4601/14/9/1034/s1, Table S1: Questionnaire for Disabilities among the Population Group of over 7 Years Old.
