# Supplementary material for: Prevalence, Causes and Social Factors of Visual Impairment among Chinese Adults: Based on a National Survey
Source: Int J Environ Res Public Health. 2017 Sep 8;14(9):1034. doi: 10.3390/ijerph14091034 (PMC5615571; doi:10.3390/ijerph14091034)
Supplement: Supplementary file 1 [file ijerph-14-01034-s001.pdf]

Table S1 Questionnaire for Disabilities among the Population Group of over 7 Years Old.

**Note:**<br>1. The respondents to this questionnaire are members among the population group over 7 years;<br>2. The surveyors have at least graduated from senior high school;<br>3.The surveyor are not allowed to add or cut down any part of the content of the questionnaire so as to keep its trueness;<br>4. The surveyors must ask the respondents the following 6 questions one by one. If there is any question, please select "Yes" and fill in the Screening Delivery Receipt.<br><br>**Instruction:**<br>We will ask you several questions on the health condition of you and your family members, please cooperate with us!
1.Do you or any of your family members has eyesight problem? (Can not see clearly or can not see at all)?<br>Yes
2. Do you or any of your family members has hearing problem? (Can he or she answer your question when asking from behind)?<br>Yes
3. Do you or any of your family members has any problem in verbal communication, i.e. cannot speak, speak unclearly and have difficulty in verbal communication?<br>Yes
4. Do you or any of your family members has difficulty in:<br>（1）Walking, standing, sitting or climbing upstairs?<br>（2）Holding, writing, washing or dressing by hand in daily life?<br>Yes
5. Do you or any of your family has any members who<br>（1）is unable to finish normal learning activities (being late for school, having a poor score,, failing to go up to the next grade, or falling out of school midway)?<br>（2）Needs help because of poor living capability or poor working capability?.<br>Yes
6. Do you or any of your family has any problem bellow:<br>（1）Has a poor memory (forgetful)?;<br>（2）Has difficulty in concentration (easy to wander off)?;<br>（3）Has difficulty in controlling their moods (moody, too joyful or joyless)?<br>（4）Has strange behaviour that is out of the ordinary (language and behavior which can not be understood or accepted by a normal person)?<br>（5）Has been addicted to alcohol or drugs (often drinks alcohol with an empty stomach (no less than five times per week) ; or takes in excess hypnotic drugs)?<br>**For those aged from 7 to17 years old, please answer the following questions:**<br>（1）Are you/they lack of eye contact, listening but not hearing?<br>（2）Are you/they lonely with narrow interests, and often addicted monotonous and repeated activities?<br>（3）Is there any language or non-language barrier to intercommunication?<br><br>Yes
